# Food-type may jeopardize biomarker interpretation in mussels used in aquatic toxicological experimentation

**DOI:** 10.1371/journal.pone.0220661

**Published:** 2019-08-05

**Authors:** Esther Blanco-Rayón, Anna V. Ivanina, Inna M. Sokolova, Ionan Marigómez, Urtzi Izagirre

**Affiliations:** 1 CBET Research Group, Department of Zoology and Animal Cell Biology, University of the Basque Country (UPV/EHU), Leioa, Basque Country, Spain; 2 Research Centre for Experimental Marine Biology and Biotechnology (Plentzia Marine Station; PiE-UPV/EHU), University of the Basque Country, Plentzia, Basque Country, Spain; 3 Department of Biological Sciences, University of North Carolina at Charlotte, Charlotte, North Carolina, United States of America; 4 Department of Marine Biology, Institute for Biosciences and Department of Maritime Systems, Interdisciplinary Faculty, University of Rostock, Rostock, Germany; VIT University, INDIA

## Abstract

To assess the influence of food type on biomarkers, mussels (*Mytilus galloprovincialis*) were maintained under laboratory conditions and fed using 4 different microalgae diets *ad libitum* for 1 week: (a) *Isochrysis galbana*; (b) *Tetraselmis chuii*; (c) a mixture of *I*. *galbana* and *T*. *chuii*; and (d) a commercial food (Microalgae Composed Diet, Acuinuga). Different microalgae were shown to present different distribution and fate in the midgut. *I*. *galbana* (≈4 μm Ø) readily reached digestive cells to be intracellularly digested. *T*. *chuii* (≈10 μm Ø and hardly digestible) was retained in stomach and digestive ducts for long times and extracellularly digested. Based on these findings, it appeared likely that the presence of large amounts of microalgal enzymes and metabolites might interfere with biochemical determinations of mussel’s biomarkers and/or that the diet-induced alterations of mussels’ digestion could modulate lysosomal and tissue-level biomarkers. To test these hypotheses, a battery of common biochemical, cytological and tissue-level biomarkers were determined in the gills (including activities of pyruvate kinase, phosphoenolpyruvate carboxykinase and cytochrome c oxidase) and the digestive gland of the mussels (including protein, lipid, free glucose and glycogen total content, lysosomal structural changes and membrane stability, intracellular accumulation of neutral lipids and lipofuscins, changes in cell type composition and epithelial thinning, as well as altered tissue integrity). The type of food was concluded to be a major factor influencing biomarkers in short-term experiments though not all the microalgae affected biomarkers and their responsiveness in the same way. *T*. *chuii* seemed to alter the nutritional status, oxidative stress and digestion processes, thus interfering with a variety of biomarkers. On the other hand, the massive presence of *I*. *galbana* within digestive cells hampered the measurement of cytochemical biomarkers and rendered less reliable the results of biochemical biomarkers (as these could be attributed to both the mussel and the microalgae). Research to optimize dietary food type, composition, regime and rations for toxicological experimentation is urgently needed. Meanwhile, a detailed description of the food type and feeding conditions should be always provided when reporting aquatic toxicological experiments with mussels, as a necessary prerequisite to compare and interpret the biological responses elicited by pollutants.

## Introduction

Mussels are widely used sentinel organisms in pollution monitoring programs to assess the biological effects of pollutants. There is an urgent need to develop consensus standardized procedures for biomarker determinations [1,2]. Recently, a large effort has been directed towards development of the Best Available Practices for mussel sampling and processing in field studies and monitoring programs [3,4,5]. Together with field studies, laboratory experiments are crucial to gain understanding of the biological effects of pollutants and to develop a reliable toolbox of biomarkers for environmental monitoring and assessment. Some experimental variables such as temperature, photoperiod, salinity, water renewal, and dosing are recognized as key conditions to correctly perform laboratory experiments and routinely reported in publications and research reports. However, less attention has been paid to the potential effects of the food type and feeding strategy. Although digestion and food type may modulate biomarker responsiveness [6,7,8], to our knowledge, there is no guidelines dealing with recommended food types and feeding strategies to keep mussels during laboratory experiments. Thus, a large variety of food types and feeding strategies are used in aquatic toxicological experiments with mussels; these include absence of additional food supply, supply of diverse commercial food products or a variety of live microalgae either in monocultures or in mixtures. Moreover, in many cases no mention is made to the food type or feeding conditions. For example, in a non-exhaustive literature mining in which 75 classical and recent manuscripts were selected, a 16% of the papers provided no indication of whether mussels were fed during experimentation, 11% maintained mussels without additional food supply (particularly during short-term experiments), 40% of the studies used live microalgae in monoculture (29%) or in mixtures (11%), and a 33% used commercial food of diverse origins (14 manufacturers) and/or nature-derived food (such as lyophilized algae or flour) (Table 1). Moreover, the rations and regime of food availability (e.g., continuous flow vs. pulses) were also different. If, the food type and feeding strategy influence the levels and responsiveness of biomarkers, the generalizations and comparisons between these experiments would be difficult.

**Table 1:** Different diets, exposure times and stressors used in recent toxicological experiments with mussels.

Food type	Food composition /source	Time (wk)	Stress source	Biomarkers*	References
**Not reported**		<1	Cu	2	[9]
			Diclofenac	1, 2	[10]
			B[a]P	4	[11]
		1	Cu, Hg, CH_3_Hg	2	[12]
			H_2_O_2_	2, 3	[13]
		2	Drugs	2	[14]
				1, 2	[15]
		4	Chemical mixtures	2	[16]
			Cd and thermal	1, 2	[17]
		5	Cd	2	[18]
			Crude oil WAF	1	[19]
		6	Cd and thermal	2, 5	[20]
**No additional food supply**		<1	B[a]P	2	[21]
			TiO_2_ NPs	1, 2, 3	[22]
				3, 4	[23]
			PAHs	5	[24]
		1	Nickel	2	[25]
			B[a]P+ Cu	2	[26]
			Anoxia	2	[27]
		2	Cd-based QDs	4	[28]
		3	Cd	1	[29]
**Microalgae pure culture**	*Isocrhrysis galbana*	<1	Cd thermal	3	[30]
			Osmotic	4	[31]
	*Phaeodactylum tricornutum*		Thermal	3	[6]
			PAHs and chloroquine	3	
	*I*. *galbana*	1	ZnPT	2	[32]
			4-Nonylphenol	2	[33]
	*P*.*tricornutum*		Anoxia	3	[6]
			Paraquat	3	
			Cu and fasting	3	
	*Scenedesmus subspicatus*		Drospirenone	2, 4	[34]
	*I*. *galbana*	2	Thermal	2, 3, 4	[35]
			Pyrene	3, 5	[36]
	*P*.*tricornutum*		Cu	3	[6]
			Pyrene	3, 5	[36]
	*Tetraselmis sp*.		Oiled food	2	[37]
	*Chaetoceros muelleri*		Polystyrene microbeads	1, 2	[38]
	*I*.*galbana*	3	Pesticides	2	[39]
	*P*. *tricornutum*		Cu, Phen and fasting	3	[40]
	*I*. *galbana*	4	Thermal	1, 2, 3	[41]
	*Macrocystis pyrifera*		Cu	3	[42]
	*I*. *galbana*	8	Fluoranthene	2, 5	[8]
	*P*. *tricornutum*	26	Crude oil WAF	3, 5	[43]
**Microalgae mixture**	*Chrysophyta*, *T*. *chui*	<1	Cd and *Vibrio*	2	[44]
	*I*. *galbana*, *C*. *gracilis*, *T*. *suecica*	1	Saponin	2	[45]
	*Isochrysis*, *Rhodomonas*	3	Endocrine disruptors	2, 4	[46]
	*I*. *galbana*, *C*. *gracilis*, *T*. *suecica*	4	PCB153	2	[47]
	*I*.*galbana*, *R*. *baltica*, *S*.*costatum*	30	Dispersed crude oil	1, 4	[48]
**Microalgae + OM**	*C*. *neogracile*, *H*. *triquetra*	2	Fluoranthene	1, 2, 5	[49]
**Microalgae + CF**	*I*. *galbana* + SERA	3	CuO NPs	1, 2, 4	[50]
**COMMERCIAL FOOD (CF)**	Korall fluid	<1	Cd and crude oil WAF	3	[7]
			Cd and thermal	2, 3	[51]
	Liquifry		Cr (VI)	1, 2, 3	[52]
			Pesticides	2, 3	[53]
	KORAL	1	Cr (VI)	3	[54]
	Phytofeast	2	Drugs	2, 5	[55]
	Easy Reefs	3	PE Microparticles	2, 4, 5	[56]
	Hawaiian Marine Imp Inc		Crude/Lubricant oil WAF	3	[57]
			Organochemicals	2	[58]
			Organochemicals	3	[59]
	Marine Invertebrate Diet		B[a]P and Cd	3	[60]
	SERA		Fuel oil WAF	2	[61]
	Shellfish diet 1800		Cu	1, 2, 3, 5	[62]
	AlgaMac protein+	4	Carbamazepine	2, 5	[63]
	Algal feed		Cd	5	[64]
	Coast Oyster Co		PAHs and PCBs	2, 3	[65]
	Drymicroencapsules Myspat		Pesticides	1, 2	[66]
	Shellfish diet 1800	5	Treated produced water	1, 2, 3, 4	[67]
	Hawaiian Marine Imp Inc	6	Cu, Zn, Cd	4	[68]
		13	Crude /Lubricant oil WAF	4	[69]
				4, 5	[70]
				4	[71]
				3	[72]
				3	[73]
	Saunders-Microencapsulates	14	Metals (Hg, Ag, Pb, Cu)	4	[74]

* Types of biomarkers depending on their endpoint and technology: (1) Functional and in vitro assays; (2) Biochemistry and molecular biology; (3) Cryotechnology and cytochemistry; (4) Histo(path)ology; (5) Biometry and physiology.

To test the hypothesis that the feeding regime might affect the toxicologically important biomarkers, the present investigation was aimed at determining the influence of food type on a battery of biomarkers frequently analysed in mussels (*Mytilus galloprovincialis*).

As the initial step, the distribution and fate of the microalgae of different sizes and biochemical composition (*Isochrysis galbana*, *Tetraselmis chuii* and their mixture) were investigated in the mussels’ midgut by light and fluorescence microscopy following *ad libitum* feeding for 5 min, 2 h and 5 d. These algal species are commonly used as food for bivalves in aquatic toxicological experimentation (Table 1). We then exposed the mussels to different diets (*I*. *galbana*; *T*. *chuii*; *I*.*galbana* and *T*.*chuii* microalgae mixture; and commercial Shellfish Diet microalgae blend, Acuinuga) for a week and investigated a battery of biomarkers commonly employed for biological effect assessment in marine pollution monitoring. These biomarkers included activities of key metabolic enzymes (cytochrome c oxidase, pyruvate kinase, phosphoenolpyruvate carboxykinase), oxidative lesions of proteins and lipids, lysosomal membrane stability, and tissue-level markers for the integrity and health of digestive epithelia. Cytochrome c oxidase (COX) was used as a marker of mitochondrial capacity that commonly correlates with mitochondrial activity and oxygen consumption rates [75–79]. Pyruvate kinase (PK) and phosphoenolpyruvate carboxykinase (PEPCK) channel the glycolytic substrate (pyruvate) to the aerobic (PK) vs. anaerobic (PEPCK) pathways [80, 81], so that the PK/PEPCK ratio is commonly used as a measure of the relative aerobic/anaerobic capacity of the organism [81]. Increased levels of protein carbonyl groups (CO) are signs of early oxidative damage (protein oxidation) whilst increased levels of malondialdehyde (MDA) and 4-hydroxy-2-nonenal (HNE) indicate later oxidative damage (lipid peroxidation) [26,82,83]. Lysosomal enlargement and membrane destabilization in mussel digestive cells are widely used as pollution effect biomarkers [1,2,59,84]. Intracellular neutral lipid accumulation has been related to organic xenobiotic exposure, non-specific stress and nutritional status [59,85–87]. The relative proportion of basophilic cells is known to increase in the digestive gland epithelium under stress conditions [88]. Atrophy of the digestive epithelium and loss of digestive gland histological integrity occur in response to pollutant exposure [71,89–91]. Therefore, investigation of the battery of these biomarkers provided a comprehensive insight into the potential impact of the altered diet quality on the integrated metabolic and stress response of the mussels.

## Material and methods

### Experimental design and sample processing

Intertidal mussels (*M*. *galloprovincialis*) of 3.5–4.5 cm shell length were collected from the low tide-mark level (0.5–1.0 m) in Plentzia (Basque Coast; 43°26′N; 2°55′W) in September 2014. Permits by the Directorate of Fishing and Aquaculture of the Department of Economical Development and Infrastructures of the Basque Government were obtained for mussel collection in public domains of the Basque Coast (Law 6/1998; BOPV N. 62, 1/4/1998). Additional permits were not required because *M*. *galloprovincialis* is not an endangered or protected species. Mussels were acclimatized for 7 d to laboratory conditions (18±1°C; 12L:12D cycle), maintained unfed in filtered (0.2 μm) seawater (dissolved oxygen: 7.6–8.3 mg/l; pH 7.8–8; salinity: 33‰).

**Fig 1:**
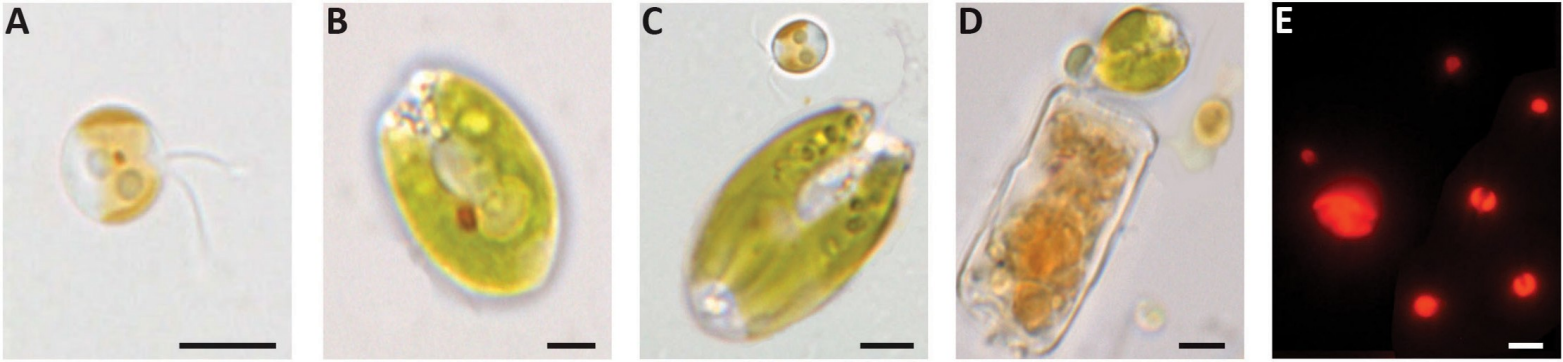
**(A-D) Appearance at the light-microscope of the various dietary food types (unstained smears).** (A) *Isochrysis galbana*; (B) *Tetraselmis chuii*; (C) mixture of *I*. *galbana* and *T*. *chuii*; and (D) commercial food. (E) Autofluorescence signal in frozen unstained smear of *I*. *galbana* (small particles) and *T*. *chuii* (large particles). Scale bar: 2 μm.

After acclimatization, mussels were divided in 4 experimental groups (in 5 l seawater tanks with constant aeration, n = 20) and fed *ad libitum* for a week using four different microalgae diets commonly used as food for mussels in laboratory experimentation: (a) *I*. *galbana*, (b) *T*. *chuii*, (c) a mixture of *I*. *galbana* and *T*. *chuii*, and (d) a commercial food (Microalgae Composed Diet, Acuinuga SL, A Coruña, Spain). *I*. *galbana* is a brown free-living biflagellate marine microalga (≈4 μm Ø; Fig 1) (Table 1). *T*. *chuii* is a green free-living tetraflagellate marine microalga (≈10 μm Ø; Fig 1) (Table 1). The used commercial food (Fig 1) is based on a mixture of 4 microalgae *Isochrysis sp*. (25%), *Tetraselmis sp*. (25%), *Thalassiosira sp*. (25%) and *Nannochloropsis sp*. (25%). Following the manufacturer recommendations the commercial food was stored at -40°C before use. Once opened it was stored at <6°C for 1 week during the experiments.

Strains of *I*. *galbana* (T. ISO clone) and *T*. *chuii* were grown in previously cleaned 30 L volume methacrylate reactors with natural filtered seawater. Monocultures were maintained under constant white light exposure (two lamps of 36 W per reactor), room temperature (T = 17°C) and filtered air flow (0.2 μm filters). Microalgae culture density was checked daily using a Beckman Coulter Counter Z2 particle size analyser, and diluted as needed in seawater enriched with F/2 medium (Easyalgae Fitoplancton Marino SL, Cádiz, Spain) to keep an average concentration (cell/mL) of 7.8±1.4×10^6^ for *I*. *galbana and* 11.4±3.4×10^5^ for *T*. *chuii*. Water and food from mussel tanks (total volume: 3 l seawater+food per tank) were changed every day: (a) 3 l/d of *I*. *galbana* culture; (b) 3 l/d of *T*. *chuii* culture; and (c) 1.5 L/d of *I*. *galbana* culture and 1.5 l/d of *T*. *chuii* culture for the mixture diet. The commercial food (2×10^9^ particles/mL; Microalgae Composed Diet) was diluted in seawater in order to provide a concentration of 5×10^6^ particles/ml in 3 l seawater for daily changes.

Immediately after the acclimatization period and after 5 min, 2 h and 1 wk feeding with *I*. *galbana* and *T*. *chuii* and their mixture, the digestive gland was dissected from several mussels, snap frozen in liquid nitrogen and stored at -80°C until further analysis. The autofluorescence of cryotome sections (8 μm) of these digestive glands was examined under the Nikon Eclipse Ni-Series fluorescence microscope using a 485 nm excitation filter and a 645 nm emission filter to visualize algal chlorophyll [92]. Schmorl's staining was applied to visualize lipofuscin in the same cryotome sections [93].

After a week of experimental exposures, gills and digestive gland of five mussels were dissected, frozen in liquid nitrogen and stored at -80°C for biochemical and histochemical analyses. Mantle and digestive gland of 10 mussels were dissected, fixed in formaldehyde (4% in seawater) at 4°C and embedded in paraffin for histological analyses. No mortality was observed during experimental exposures. Gonad histology was examined to provide supporting data of mussel general condition [94]; upon microscopic examination of mantle tissue sections all the individuals in all the treatments were found to be at a comparable gametogenic stage (Gonad Index = 1.25 ± 0.21).

### Biochemical analysis

Total lipid content was determined in the digestive gland using a chloroform extraction method [95,96]. Briefly, about 50 mg of the digestive gland tissue was homogenized in chloroform/methanol mixture (2:1 v:v) using tissue: solvent proportion of 1:20 w/v. Samples were sonicated for 1 min (output 69 W, Sonicator 3000, Misonix, Farmingdale, NY, USA), incubated overnight at 4°C and centrifuged for 5 min at 13000×*g*. The supernatant was transferred in a new tube, mixed with ultrapure water (0.25 volumes of the supernatant), vortexed for 2 min and centrifuged for 5 min at 13000×*g*. The lower phase (chloroform) was transferred into a pre-weighed microcentrifuge tube and allowed to evaporate to determine the dry mass of extracted lipids. For determination of carbohydrates, the digestive gland tissue was powdered under liquid nitrogen and homogenized with five volumes of ice-cold 0.6 M perchloric acid (PCA) with 150 mM ethylenediaminetetraacetic acid (EDTA) [97]. An aliquot of the homogenate was reserved for glycogen determination, and the remaining homogenate was centrifuged to remove precipitated protein and neutralized with 5 M potassium hydroxide to pH 7.2–7.5. Precipitated potassium perchloride was removed by a second centrifugation and extracts were stored at −80°C. Carbohydrates were measured in neutralized PCA extracts using a standard NADPH-linked spectrophotometric test [98]. Briefly, assay conditions were as follows: 38.5 mM triethanolamine buffer, pH 7.6, 0.04 mM NADP^+^, 7 mM MgCl_2_·6H_2_O, 0.462 U/ml glucose-6-phosphate dehydrogenase, 1.8 U /ml hexokinase. Glycogen concentration was measured in PCA extracts after enzymatic hydrolysis of glycogen to D-glucose by glucoamylase [99] and determined by the difference in the D-glucose levels in the tissue extract before and after glucoamylase treatment. Concentrations of glycogen, lipids and proteins were expressed in mg/g wet tissue mass.

For total protein content analysis, digestive gland was homogenized in ice-cold homogenization buffer (100 mM Tris, pH 7.4, 100 mM NaCl, 1 mM EDTA, 1 mM egtazic acid (EGTA), 1% Triton-X 100, 10% glycerol, 0.1% sodium dodecylsulfate, 0.5% deoxycholate, 0.5 μg leupeptin/ml,0.7 μg pepstatin/ml, 40 μg phenylmethylsulfonyl fluoride (PMSF) /ml and 0.5 μg /ml aprotinin) using Kontes Duall tissue grinders (Fisher Scientific, Suwanee, GA, USA). Homogenates were sonicated 3×10 sec each (output 69 W, Sonicator 3000, Misonix), with cooling on ice between sonications, centrifuged for 10 min at 20000 *g* and 4°C, and supernatants were used for protein determination. Protein content was measured using the Bio-Rad Protein Assay kit according to the manufacturer's protocol (Bio-Rad Laboratories, Hercules, CA, USA).

Activities of the pyruvate kinase (PK; EC 2.7.1.40), phosphoenolpyruvate carboxykinase (PEPCK; EC 4.1.1.31) and cytochrome c oxidase (COX; EC 1.9.3.1) were determined in the gills. The tissues were homogenized in enzyme-specific homogenization buffer using hand-held Kontes Duall tissue grinders (Fisher Scientific, Suwanee, GA, USA). Homogenates were sonicated 3×10 sec each (output 7, Sonic Dismembrator Model 100, Fisher Scientific, Suwanee, GA) to ensure complete release of the enzymes, with cooling on ice (1 min) between sonications and centrifuged at 16000×*g* and 4°C for 25 min. The supernatant was collected and used for enzyme determination. Enzyme extracts were stored at −80°C for less than two weeks before activity assays. For determination of enzyme activities, enzyme extracts were thawed on ice and immediately analyzed by standard spectrophotometric techniques as described elsewhere [98,100,101] using a UV–Vis spectrophotometer (VARIAN Cary 50 Bio, Cary NC, USA). The temperature of the reaction mixture was controlled at 20±0.1°C using a water-jacketed cuvette holder. Briefly, isolation and assay conditions for the studied enzymes were as follows: (a) PK: homogenization buffer: 10 mM Tris–HCl buffer (pH 7.2), 5 mM EDTA, 1 mM dithiotreitol (DTT), 0.1 mM phenylmethylsulfonyl (PMSF); assay: 50 mM Tris-HCl (pH7.2), 50 mM KCl, 5 mM MgSO_4_, 1 mM ADP, 0.2 mg/ml NADH, 5.5 U/ml LDH, 0.5 mM phosphoenolpyruvate (PEP); acquisition wavelength: 340 nm; (b) PEPCK: homogenization buffer: 10 mM Tris–HCl buffer (pH 7.2), 5 mM EDTA, 1 mM DTT, 0.1 mM PMSF; assay: 100 mM 4-(2-hydroxyethyl)-1-piperazineethanesulfonic acid (HEPES) (pH 7.2), 2.3 mM MnCl_2_, 0.5 mM Inosine-5’- diphosphate trisodium salt (IDP), 5mg/ml KHCO_3_, 0.2 g/ml NADH, 10 U/ml malate dehydrogenase (MDH), 15 mM PEP; acquisition wavelength: 550 nm; (c) COX: homogenization buffer: 25 mM potassium phosphate, pH 7.2, 10 μg/ml PMSF, 2 μg/ml aprotinin; assay: 20 mM potassium phosphate, pH 7.0, 16 μM reduced cytochrome c(II), 0.45 mM n-dodecyl-b-d-maltoside, 2 μg/ml antimycin A; acquisition wavelength: 550 nm. Protein concentration was measured as above described for the digestive gland.

Protein carbonyl groups (CO) were measured spectrophotometrically [102]. Digestive gland was ground under liquid nitrogen and homogenized in buffer containing 50 mM HEPES, 125 mM KCl, 1.1 EDTA and 0.6 mM MgSO_4_ (pH 7.4) and protease inhibitors [leupeptin (0.5 μg/ml), pepstatin (0.7 μg/ml), phenylmethylsulfonyl fluoride (40 μg/ml) and aprotinin (0.5 μg/ml)]. Samples were centrifuged at 100000×*g* for 15 min, supernatant was collected and incubated at room temperature with 10 mM 2,4-dinitrophenylhydrazine (DNP) in 2 M HCl. The blanks were incubated with HCl without DNP. After incubation, proteins were precipitated by adding 100% trichloracetic acid and centrifuged at 11000×*g* for 10 min. The pellet was washed with ethanol ethylacetate (1:1 v:v) and resuspended in 6 M guanidine hydrochloride in 20 mM in KH_2_PO_4_ (pH 2.5) until dissolved. The absorbance was measured at 360 nm on a spectrophotometer (VARIAN Cary 50 Bio, Cary NC, USA) using guanidine HCl solution as reference. The amount of carbonyls was estimated as a difference in absorbance between samples and blanks using a molar extinction coefficient of carbonyls ɛ = 22000 1/(cm×M). Protein content of the samples was determined using BSA standard prepared in 6 mol/l guanidine HCL and 20 mmol /L KH_2_PO_4_ (pH 2.4). Carbonyl content was normalized to the protein concentration in the samples.

Protein conjugates of malondialdehyde (MDA) and 4-hydroxynonenal (4-HNE) were measured as biomarkers of lipid peroxidation using enzyme-linked immunosorbent assay (MDA OxiSelect MDA adduct ELISA Kit and HNE OxiSelect HNE-His adduct ELISA Kit, respectively) according to the manufacturers' protocols (Cell Biolabs, Inc., CA, USA). About 200–300 mg of digestive gland were homogenized in ice cold phosphate-buffered saline (PBS) (1:5 w:v) with protease inhibitors (50 μg/l aprotinin and 40 μM phenylmethylsulfonyl fluoride) using Kontes Duall tissue grinders (Fisher Scientific, Suwanee, GA, USA). Samples were centrifuged at 15000×*g* for 10 min at 4°C. Protein concentration was measured in the supernatant using the Bio-Rad Protein Assay kit according to the manufacturer's protocol (Bio-Rad Laboratories, Hercules, CA, USA). Supernatants were diluted with PBS to a final concentration of 1 mg/l protein.

### Histological and histochemical analyses

Digestive gland sections (5 μm thick) were cut in a Leica RM2125 microtome, and mounted on albumin coated slides, dried at 37°C for 24 hr, and stored at room temperature until staining with toluidine-eosin [103]. Sections were dewaxed in xylene, rehydrated in serial dilutions of alcohols followed by distilled water. The rehydrated sections were rinsed in 1% toluidine in distilled water for 10 min, followed by the tap water. The sections were then rinsed in eosin for 15 sec, washed in tap water, dehydrated in ascending graded-ethanol series, cleared in xylene and mounted in DPX. A stereological procedure was used to quantify the volume density of basophilic cells (Vv_BAS_), the mean epithelial thickness (MET; μm) and the mean luminal radius (MLR; μm) and to measure the connective tissue-to-diverticula ratio (CTD) was also calculated [104]. Counts were made in 3 optical fields per mussel in 6 mussels per experimental group. Slides were viewed at 40× magnification using a drawing tube attached to a Nikon Eclipse Ni microscope. A Weibel graticule (multipurpose system M-168) was used and hits of basophilic and digestive cells, luminal area and connective tissue were recorded to calculate Vv_BAS_, MLR/MET and CTD [67,91].

Lysosomal membrane stability was evaluated in serial cryotome sections (10 μm thick; Leica CM3050S cryotome) in the digestive glands of individual mussels (5 per experimental treatment) after the cytochemical demonstration of hexosaminidase activity, according to a standardised procedure [1], based on the time of acid labilisation (LP) required to produce the maximum staining intensity. LP was determined at the light microscope as the maximal accumulation of reaction product associated with lysosomes. Four determinations were made per digestive gland and their mean value corresponded to an individual digestive gland LP, expressed in min.

To quantify changes in lysosomal structure, digestive gland cryotome sections (8 μm thick) from the digestive glands of individual mussels (5 per experimental treatment) were stained for the histochemical demonstration of ß-glucuronidase activity [57]. Five measurements using a 1000× magnification were made in each section using image analysis (Sevisan S.L., Spain).The values of the following stereological parameters were determined and averaged between the five replicate measurements in each mussel digestive gland [105]: lysosomal volume density (Vv_LYS_ = V_LYS_/V_C_), lysosomal surface density (Sv_LYS_ = S_LYS_/V_C_), lysosomal surface to volume ratio (S/V_LYS_ = S_LYS_/V_LYS_) and lysosomal numerical density (Nv_LYS_ = N_LYS_/V_C_, where V is volume, S is surface, N is number, LYS is lysosomes and C is the cytoplasm of the digestive cell.

Intracellular neutral lipids accumulation was determined in cryotome sections (8 μm thick) of the digestive glands (N = 5 per experimental treatment). The neutral lipids were visualized by by staining with Oil Red O (ORO) [59]. Slides were viewed at 400× magnification. The extent of ORO staining in the digestive gland epithelium was measured using image analysis as described elsewhere [35,67]. The volume density of ORO positive reaction product (neutral lipids) with respect to the digestive epithelium volume (Vv_NL_) was calculated by applying a stereological procedure [59]. Vv_NL_ is expressed as μm^3^/ μm^3^.

Lipofuscin (LPF) accumulation was determined in digestive gland cryostat sections (8 μm thick) fixed for 15 min in Baker buffer at 4°C. The sections were rinsed in distilled water and stained using Schmorl's reaction [93]. Five measurements using a 400× magnification were made in each section using image analysis (Sevisan S.L., Spain). The mean value of LPF volume density (Vv_LPF_ = V_L_/V_C_) was determined for each mussel digestive gland (N = 5 per experimental treatment).

### Statistical analysis

Statistical analyses were made using SPSS v 22.0 software (SPSS INC., Chicago, Illinois). Parameters were tested for normality (Kolmogorov-Smirnov's test) and homogeneity (Levene's test). For the traits that had normal distribution and homogeneous variances (COX, PK, PEPCK, PK/PEPCK, MDA, HNE, Vv_NL_, Vv_LPF_ Vv_BAS_, MLR/MET and CTD), one-way ANOVA and Duncan's *post-hoc* tests were used to test for the effects of the diet type and conduct the pairwise comparisons of group means, respectively. For LP, non-parametric statistics (Mann-Whitney's U-test) was used. The Z-score test was used when the sample size was too small (N = 4) for reliable Duncan's or Mann-Whitney's U-test including the following traits: total lipid content, glycogen, total protein content, Vv_LYS_, S/V_LYS_, and Nv_LYS_. Significance for all statistical tests was established at p<0.05.

## Results

### Microalgae distribution and fate in the midgut

**Fig 2:**
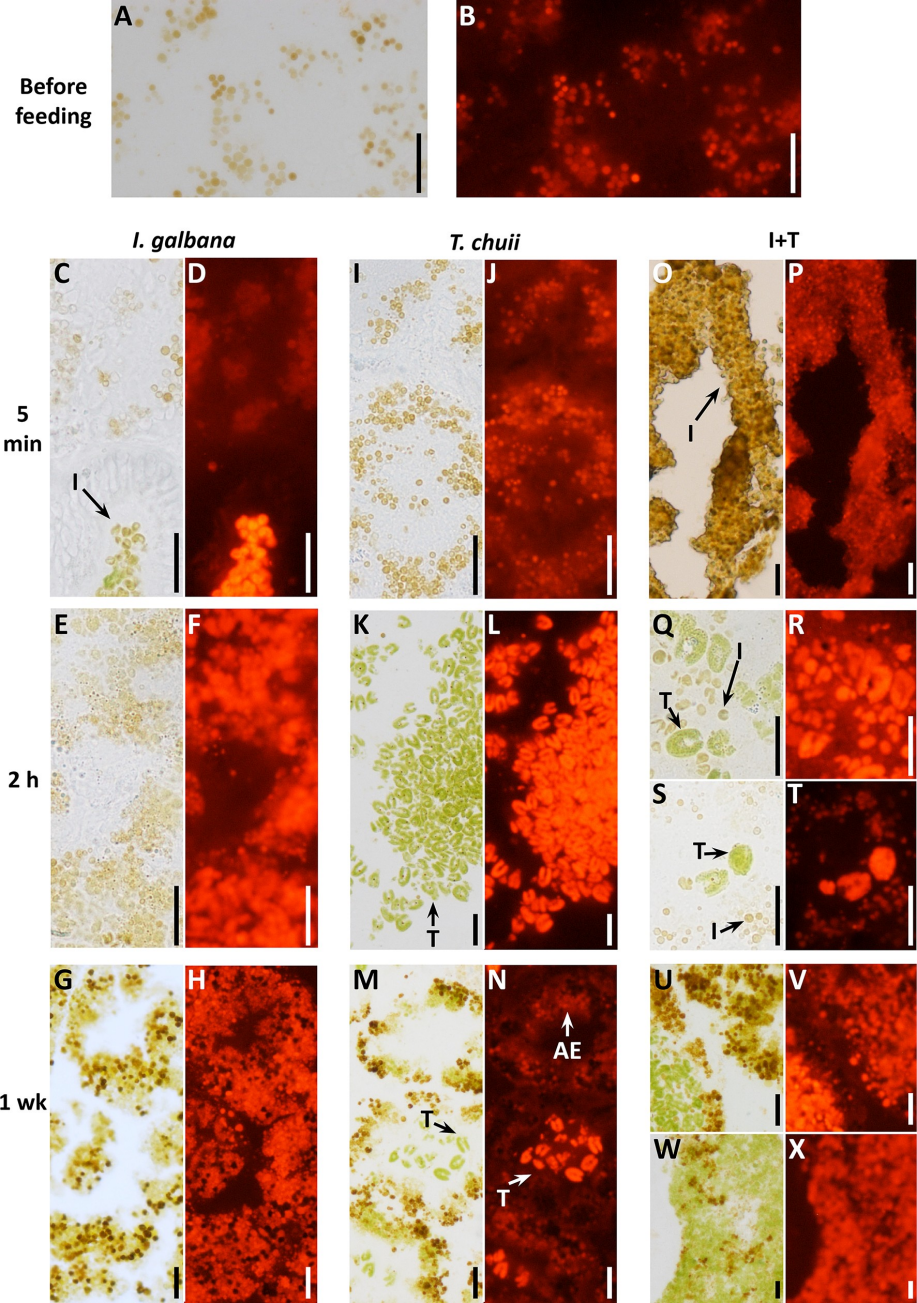
**Cryostat sections (8 μm) of unstained fresh tissue of mussels:** (A, C, E, G, I, K, M, O, Q, S, U, W) Before feeding and after *ad libitum* feeding for 5 min, 2 h and 1 week with *I*. *galbana*, *T*. *chuii* and *I*. *galbana* + *T*. *chuii*; (B, D, F, H, J, L, N, P, R, T, V, X) The same tissue section fields examined at the fluorescence microscope with 485 nm excitation filter and 645 nm emission filter. B, D and F: 12% light intensity; H: 6% light intensity. J, L, N, P, R, T, V and Y: 3% light intensity. Scale bar: 20 μm. I, *I*. *galbana*-like body; T, *T*. *chuii*; AE, alveolus epithelium.

**Fig 3:**
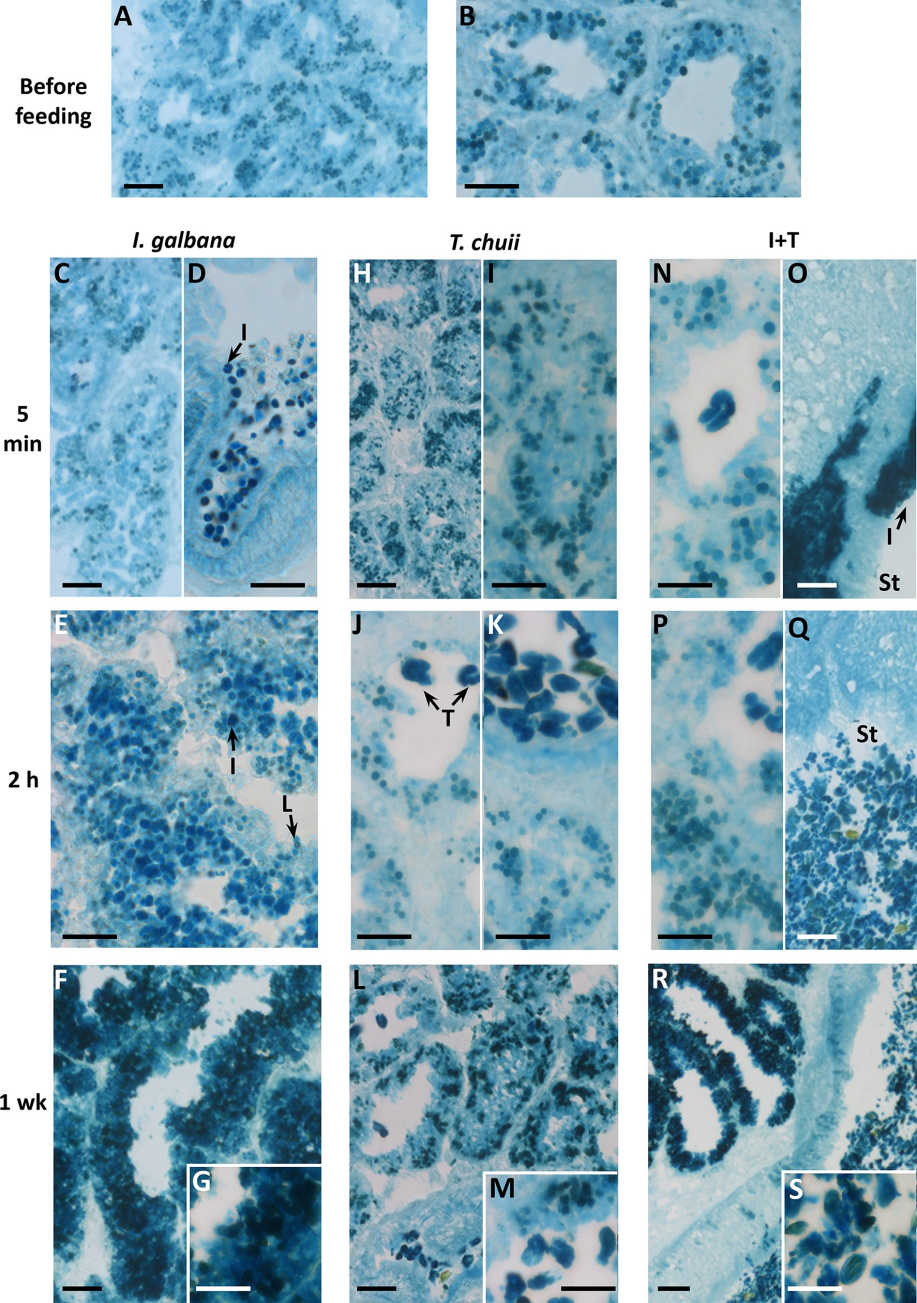
Histochemistry of lipofuscins in digestive gland of mussels, before feeding and after feeding *ad libitum* with *I*. *galbana*, *T*. *chuii* and *I*. *galbana* + *T*. *chuii* for 5 min, 2 h and 1 wk. Scale bar is 30 μm in A, C, F, H, Q, L, O);. 20 μm in B, D, E, G, I, J, K, M, N, P, S); and 50 μm in S. I, *I*. *galbana*-like body; T, *T*. *chuii*; St, stomach; L, lipofuscin.

After 7 days of acclimatization without food, some brownish granules were observed in the lumen and in the epithelium of digestive alveoli (Fig 2A). These granules exhibited weak fluorescence (Fig 2B) and were identified as LPFs (Fig 3A and 3B), likely related to residual bodies of digestive cells.

Similar fluorescent LPF-like granules were observed after feeding mussels with *I*. *galbana* for 5 min (Figs 2C, 2D, 3C and 3D). Microalgae were found in the lumen of the stomach as well as in primary and secondary digestive ducts (Fig 2C). These microalgae presented a strong fluorescence (Fig 2D). In contrast, no microalgae were found, nor fluorescence detected in the digestive alveoli (Fig 2C and 2D). Although microalgae pigments also stained with the Schmorl's method, they were easily distinguishable from the mussels’ LPFs because of the different morphology and much higher staining intensity of the microalgae (Fig 3C and 3D). After 2hr, microalgae-like bodies were observed within the epithelium of digestive alveoli (Fig 2E), which exhibited a remarkable fluorescence intensity (Fig 2F). After 1 week of feeding with *I*. *galbana*, abundant dark brown bodies were found in the epithelium of digestive alveoli together with yellowish corpuscles resembling microalgae (Fig 2G). Schmorl-positive materials (both LPFs and microalgae, which were indistinguishable due to the high intensity of the Schmorl's reaction) were extremely abundant (Fig 3F and 3G) and fluorescence intensity increased throughout the epithelium, although some small patchy areas with apparent LPFs appeared dark (Fig 2H).

After feeding mussels *ad libitum* with *T*. *chuii* for 5 min, Schmorl-positive brownish bodies with background weak fluoresce were found (Figs 2I, 2J, 3H and 3I), similar to those found after 7 days of starvation during acclimatization (Fig 2A, 2B, 2I and 2J). In contrast, after 2 h of feeding, abundant microalgae and their large fragments were found in the stomach but not in the digestive alveoli (Fig 2K). These microalgae exhibited intense fluorescence (Fig 2L) and were highly reactive after Schmorl`s staining (Fig 3J and 3K). No change was observed in the reactivity of the alveolus epithelium after Schmorl's staining (Fig 3J and 3K). After 1 week, highly fluorescent microalgae and their fragments were found in the lumen of alveoli, but not in the epithelium (Fig 2M and 2N). In contrast, the amount and staining intensity of LPFs in the epithelium of digestive alveoli increased (Fig 3L and 3M).

In mussels fed with the mixture of both microalgae species (I+T) for 5 min, a compact highly fluorescent mass was found in the stomach lumen in which some bodies resembling *I*. *galbana* could be identified (Fig 2O and 2P). After 2 h, both microalgae species were found in the lumen of the stomach (Fig 2Q and 2R) and of the alveoli (Fig 2S and 2T). After 1 week, the stomach lumen was full of microalgae and microalgae fragments (Fig 2W and 2X), with a strong fluorescence (Fig 2V and 2X). However the appearance of digestive alveoli was diverse. Some alveoli with empty lumen presented high fluorescence intensity in their epithelium (Fig 2V) whilst other had the lumen full of *T*. *chuii* and fragments, and exhibited low fluorescence within the epithelium (Fig 2U and 2V). Overall, the LPF content increased greatly in the epithelium of the digestive alveoli after 1 week of feeding with the algal mixture (Fig 3R).

### Food type influence on biomarkers

While the total protein levels were similar in all diets, the total lipid content was higher and the glycogen levels lower in *I*. *galbana* than in the other diets (Table 2). Likewise, the total protein levels were similar in all experimental mussels irrespective of the diet, while the total lipid content was lower and the glycogen levels higher in the digestive gland of mussels fed commercial food compared with other experimental groups (Table 2).

**Table 2:** Total lipid, protein and glycogen content of the food and experimental mussels fed different diets. Gross estimates for different microalgae diets (*I*. *galbana*; *T*.*chuii*; mixture of *I*. *galbana* and *T*. *chuii*; and commercial food) are based on the reports by Albentosa et al. (1996), FAO (2004) and Acuinuga Product Sheet. Values for *M*. *galloprovincialis* are based on the mussels fed *ad libitum* for 1 week on the respective diets. Letters in superscripts indicate significant differences between groups of dietary food type according to the Z-score test (p<0.05).

	Total lipids		Proteins		Carbohydrates	
	Food (% organic matter)	Mussel (g lipid/g tissue)	Food (% organic matter)	Mussel (g protein/g tissue)	Food (% organic matter)	Mussel (*mmol glycosyl unit/L)
***I*. *galbana***	30^a^	0.40±0.11^a^	20	0.95±0.19	20^a^	6.12±2.46^a^
***I*. *galbana + T*. *chuii***	20**	0.37±0.17^a^	17.5**-	0.68±0.10	35**-	7.68±5.48^a^
***T*. *chuii***	10^b^	0.32±0.09^a^	15	0.86±0.48	50^b^	1.62±1.12^a^
Commercial food***	10^b^	0.26±0.12^b^	20	0.72±0.14	45^b^	19.80±10.66^b^

* Glycogen levels

** Estimated as the average between *I*. *galbana* and *T*. *chuii* (1:1)

*** Estimates, according to the product label, subject to some variability among batches

**Fig 4:**
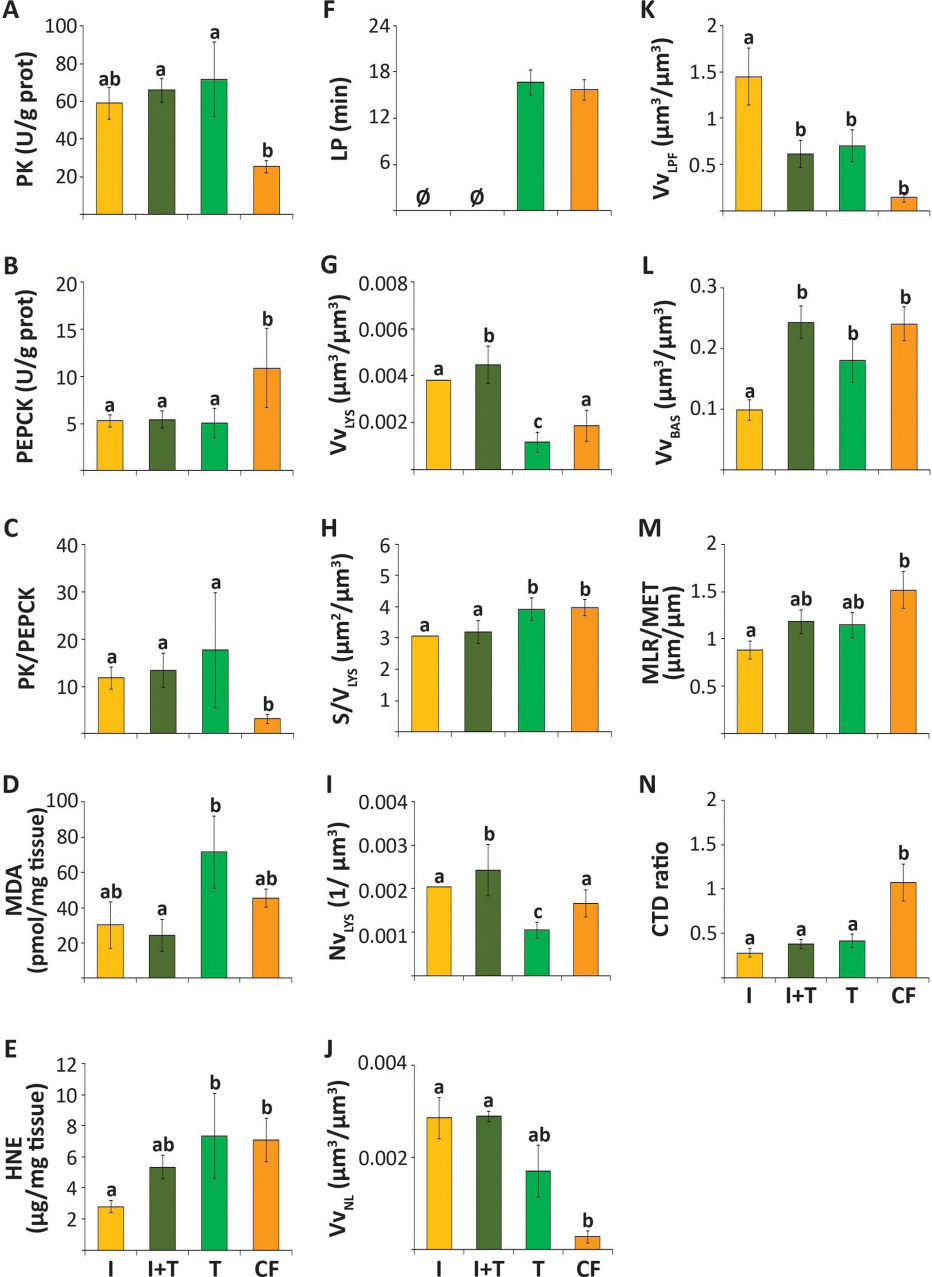
**Biomarkers recorded in mussels fed *ad libitum* for 1 week with 4 different diets** (*I*. *galbana* (I); *T*. *chuii* (T); mixture of *I*. *galbana* and *T*. *chuii* (I+T); and commercial food (CF)): pyruvate kinase (PK) (A), and phosphoenolpyruvate carboxykinase (PEPCK) (B) activities, and PK/PEPCK ratio (C) in gills; malondialdehyde (MDA)-protein conjugates (D), 4-hydroxynonemal (HNE)-protein conjugates (E), labilization period (LP) of the lysosomal membrane (F), lysosomal volume density (Vv_LYS_) (G), surface-to-volume ratio (S/V_LYS_) (H) and numerical density (Nv_LYS_) (I), volume density of neutral lipids (Vv_NL_) (J) and lipofuscins (Vv_LPF_) (K), volume density of basophilic cells (Vv_BAS_) (L), mean-luminal-radius-to-mean-epithelial-thickness (MLR/MET) (M) and connective-to-digestive-tissue (CTD) ratio (N) in digestive gland. Intervals indicate standard error. Groups labelled with a different letter are significantly different (p<0.05) from each other according to the Duncan's test performed after one-way ANOVAs except for F-I. (F-I) Different letters indicate significant differences (p<0.05) among diets according to the Mann-Whitney's U-test for LP and the Z-score test for CO, Vv_LYS_, S/V_LYS_ and Nv_LYS_. Ø, no reliable measurement.

Most studied biomarker values differed between the groups of mussels fed with the different diets (S1 Table). PK activity was lower in mussels fed commercial food than in any other experimental group (Fig 4A; S1 Table) whereas PEPCK was much higher (Fig 4B), thus resulting in a low PK/PEPCK ratio (Fig 4C). The response profiles of MDA and HNE were similar, with higher values in mussels fed *T*. *chuii* and commercial food, especially in MDA (Fig 4D and 4E; S1 Table). The CO values and COX activity were not significantly different between the treatments (S1 Fig; S1 Table).

**Fig 5:**
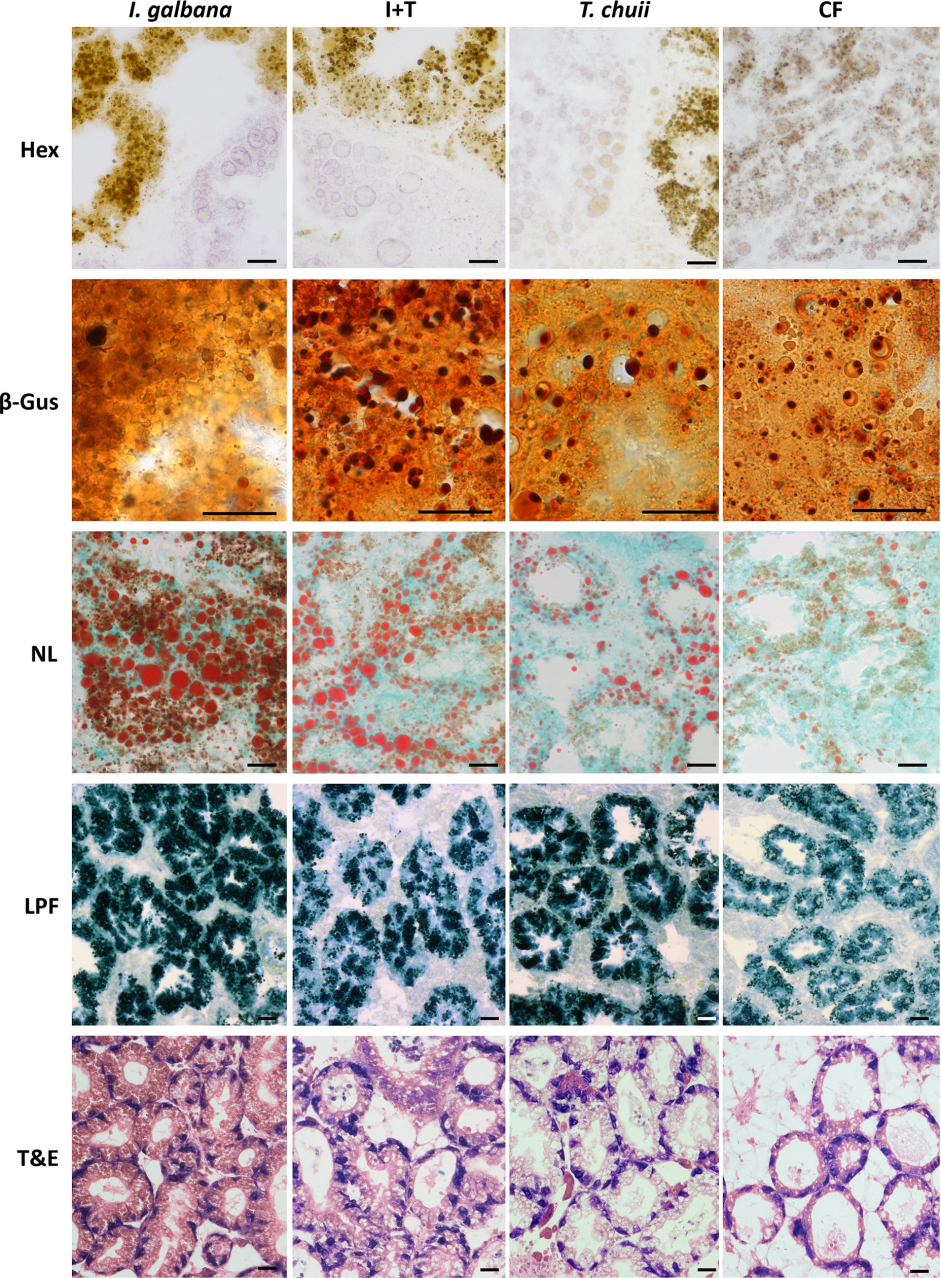
Micrographs of digestive gland of mussels fed *ad libitum* for 1 week with 4 different microalgae diets (*I*. *galbana*; *T*. *chuii*; mixture of *I*. *galbana* and *T*. *chuii* (I+T); and commercial food (CF)): hexosaminidase (Hex) and β-glucuronidase (β-gus) enzyme histochemistry, Oil Red O (neutral lipids: NL) and Schmorl's (lipofuscins: LPF) histochemistry and toluidine-eosine staining (T&E) topographical staining. Scale bar: 20 μm.

LP could not be determined in mussels fed *I*. *galbana* alone or in mixture with *T*. *chuii* because histochemical hexosaminidase activity in digestive alveoli was not clearly discriminated from the background brownish coloration caused by LPFs and microalgae (Hex in Fig 5). LP values between 15 and 20 min were recorded in mussels fed *T*. *chuii* and commercial food (Fig 4F), although in the former the measurements were difficult due to the presence of extensive brownish bodies in the digestive cells. Similarly, lysosomal structural changes (Vv_LYS_ and Nv_LYS_) were difficult to measure in mussels fed *I*. *galbana* due to the massive amount of microalgae within the epithelium of digestive alveoli (β-Gus in Fig 5. With this caveat, Vv_LYS_ was higher in mussels fed *I*. *galbana* and I+T than in those fed *T*. *chuii* or commercial food (Fig 4G; S1 Table). In contrast, S/V_LYS_ and Nv_LYS_ values did not differ among experimental groups, according to 1-way ANOVA (S1 Table). Vv_NL_ was higher in mussels fed *I*. *galbana* and I+T than in mussels fed *T*. *chuii* or commercial food, with the lowest values being in the latter group (Fig 4J; NL in Fig 5; S1 Table). Vv_LPF_ was the highest in mussels fed *I*. *galbana* and the lowest in the mussels fed commercial food, with intermediate values in the mussels fed *T*. *chuii* and the mixture of microalgae (LPF in Fig 5; Fig 4K; S1 Table).

Vv_BAS_ was lower in mussels fed *I*. *galbana* than in other experimental groups and the highest in mussels fed I+T and commercial food (Fig 4L; T&E in Fig 5; S1 Table). The lowest MLR/MET values were found in the mussels fed *I*. *galbana* and the highest in those fed commercial food (Fig 4M; T&E in Fig 5; S1 Table). CTD ratio was higher in mussels fed commercial food than in the other experimental groups (Fig 4N; S1 Table).

## Discussion

### Microalgae distribution and fate in the midgut

Microalgae species used as a food source for bivalves can differ in cell size and morphology, digestibility, biochemical composition and toxicity. Some microalgae may have a high nutritional value and be readily ingested by mussels, yet this does not necessarily imply that they will be subject to digestion [106]. For instance, after testing ten species of microalgae, only two species (*Isochrysis* and *Pavlova*) were digested by winged pearl oyster larvae [107].

After 5 min of feeding, strongly fluorescent *I*. *galbana* reached the lumen of the stomach and digestive duct of the mussels and were inside the digestive cells after 2 h of feeding or later. This finding agrees with the reported length of the digestion cycle (~ 4 h) in the intertidal mussels and the findings that *I*. *galbana* is internalized (phagocytosed) in digestive cells for intracellular digestion [3]. Similarly, digestion of *Isochrysis* sp. by giant clam veliger larvae was observed 2 h after the start of feeding [108]. With the exception of a previous study [109], the massive presence of microalgae-like spherical bodies in mussel digestive cells has not been previously reported in laboratory experiments which used *I*. *galbana* as food [36,41] even though several of these studies were based on microscopic observations of digestive gland tissue sections. Therefore, further research is needed in order to understand the mechanism through which small and relatively easily digestible microalgae such as *I*. *galbana* are digested in bivalves.

Unlike *I*. *galbana*, *T*. *chuii* took 2 h to reach the stomach and was never found within digestive cells. Similarly, digestion of *Tetraselmis* sp. by giant clam veligers was only observed at time periods exceeding 4–8 h after feeding [108]. Although *T*. *chuii* reached the lumen of digestive alveoli of the mussels and the digestive cells were rich in lipofuscins after 1 week of feeding, the epithelium was weakly fluorescent. Fluorescence intensity decays as the degree of lysis and digestion of phytoplankton cells increases [110]. Therefore, *T*. *chuii* digestion appears to be mainly extracellular and subject to extended gut retention times. Gut retention time (and associated absorption efficiency; [111]) are determined by the amount and quality, and most importantly, by the digestibility of the ingested food [112,113]. In adult oysters and mussels fed *Tetraselmis*, the gut retention time can go beyond 10 h [113]. Similarly, mussels have more difficulties in absorbing *Tetraselmis*, compared with other microalgae, and the absorption efficiency of *Tetraselmis* is half of that recorded for *Isochrysis* [106]. The large cell size can hamper absorption [107].

The presence of refractory cell walls is another potential cause for indigestibility as shown for chlorophytes in bivalves [112]. Cell walls can contain highly refractory components resistant to enzymatic attack and strong acid degradation, so that there might be little advantage in a more prolonged retention of such cells in the gut [106,112,114]. The digestibility of the cell wall of *Tetraselmis* appears low in mussels, reflected in low absorption efficiency [106]. *Tetraselmis* cells are not easily digested due to their thick cellulose-rich cell wall, which renders intracellular starch granules and other components unavailable to the gut digestive enzymes [115]. Cellulase is a common molluscan enzyme; however, hydrolysis of structural cellulose is generally low in bivalves [116]. Moreover, the cell wall of *Tetraselmis* is made of a pectin-like material, with galactose, galacturonic acid and unusual 2-keto sugar acids as major components, which make the cells walls acidic and difficult to degrade [117]. As a result, *Tetraselmis* is known to be less nutritious that *I*. *galbana* in a variety of bivalve species [115].

It is worth noting that absorption in bivalves' gut is not intestinal but depends on endocytosis and phagocytosis in digestive cells and further intracellular food digestion and nutrient delivery to haemocytes. Whilst the entire cells of *I*. *galbana* appeared taken into the digestive diverticula, in the case of *T*. *chuii* only food materials derived from extracellular digestion would be taken up by digestive cells (and lead to residual products of digestion such as lipofuscins). Interestingly, the presence of *I*. *galbana* seemed to facilitate the distribution of *T*. *chuii* towards digestive alveoli, reflected in the intermediate distribution profile of the algal cells in mussels fed with the mixture of *I*. *galbana* and *T*. *chuii* compared with the single-species diets.

In summary, different microalgae show different distribution and fate in mussel digestive gland. Whereas small microalgae such as *I*. *galbana* readily reach digestive alveoli and are intracellularly digested (albeit the extracellular pre-digestion cannot be discarded), large and hardly degradable *T*. *chuii* are retained in the stomach and digestive ducts. As a result of the presence of microalgae in the gut and digestive gland epithelium, the enzyme activities and metabolites (e.g. pigments and lipofuscins) of the microalgae or resulting from the mussel response to the microalgae can influence the determination of biochemical biomarkers. Furthermore, due to the long retention times and extracellular digestion of large microalgae, the algae-gut interactions might affect the morphology and function of the digestive cell lysosomes and thus influence lysosomal and tissue-level biomarkers in the digestive gland epithelium.

### Food type influence on biomarkers

The nutritional condition of mussels varied significantly depending on the diet. Lower total lipid content in the digestive gland of mussels fed commercial food than in those fed live microalgae reflects the relative lipid content of the diet. However, the glycogen levels in the digestive gland of mussels fed *T*. *chuii* were much lower despite the similar carbohydrate levels in the commercial food and in *T*. *chuii*, possibly reflecting the lower digestibility and absorption efficiency of *T*. *chuii*. A large part of the carbohydrates determined in *T*. *chuii* would correspond to cellulose and pectin-like material [115,117], which remained in the gut lumen and did not contribute to the carbohydrates found in the digestive gland. Overall, the commercial food (poorest diet) and *I*. *galbana* (richest diet) represented the two extreme nutritional conditions, as envisaged in the basic biochemical components measured in the digestive gland of mussels. The low digestibility of freeze-dried microalgae used in commercial food has been shown to cause reduced growth rates of bivalve seed in comparison with fresh microalgae diets [118]. Accordingly, the most striking differences in biomarker values were found when commercial food and *I*. *galbana* were compared, with the intermediate values in the mussels fed *T*. *chuii* and the *I*. *galbana*+*T*. *chuii* mixture. This finding is consistent with the earlier reports that the nutritive condition can strongly affect the biomarker values in mussels [8], albeit in this latter study the quantity rather than the quality of the food was manipulated.

Overall, differences between the groups of mussels fed different diets were found for most biomarkers investigated in our present study. Thus, PK/PEPCK was much lower in mussels fed commercial food than in those fed live microalgae, as the result of low PK and high PEPCK activities, indicating decreased aerobic scope and increased gluconeogenesis [119–123]. Lipid peroxidation (indicated by high MDA and HNE values; [26,82] was enhanced in mussels fed *T*. *chuii* or commercial food. Likewise, intracellular digestion was reduced (low Vv_LYS_ and Nv_LYS_ and high Vv_BAS,_ MLR/MET and CTD ratio; [3,7,35]) and the levels of neutral lipids (indicative of nutritional status; [87]) and lipofuscins (residual product of lipid digestion or oxidation; [83]) were low in mussels fed the commercial food. This profile might reflect lower nutritional status in mussels fed commercial food and to a lesser extent, in mussels fed *T*. *chuii*, which is known to affect biomarkers and biomarker responsiveness [49].

Furthermore, the presence of the food particles, which may vary depending on the food type and regime, may interfere with the measurement of the biomarkers. For instance, LP could not be determined in mussels fed *I*. *galbana* alone or in mixture with *T*. *chuii* because the hexosaminidase activity used to visualize lysosomes in digestive cells could not be easily discriminated against the background of brownish coloration of the lipofuscins and microalgae pigments. Likewise, Vv_LYS_ and Nv_LYS_ could not be reliably measured in mussels fed *I*. *galbana* due to the presence of massive amounts of microalgae within digestive cells, which also hampered any distinction between microalgae and lipofuscins. More subtly, biochemical determinations (e.g. MDA and HNE in digestive gland) might include the contamination with the algal-derived products potentially biasing the assessment of these biomarkers in the mussel digestive gland tissue. Future studies are needed to determine alternative experimental and analytical approaches to mitigate or reduce the diet-induced bias in biomarkers and their assessment. Furthermore, the effects of the nutritional condition and the diet must be taken into account in the biomarker assessment, as the potential effects of the nutrition are likely to be pervasive and not limited to a single tissue type.

## Conclusions

According to the present study, Best Available Practices for biomarker-based toxicological experiments should include the appropriate selection and reporting of the food type and feeding regime to achieve reliable and comparable experimental data on the biological effects of pollutants. Commercial food based on frozen or freeze-dried diets might not be the best option for feeding during toxicological experiments, similar to what was earlier shown for aquaculture production [106,118]. Live commercial phytoplankton might be a viable alternative, yet the dietary microalgae should be selected on the basis of the suitable dimension (size, volume, weight) of algal cells, high digestibility and balanced nutritional value [106]. Furthermore, different live microalgae affect biomarkers in different ways. *T*. *chuii* that has low digestibility and long gut retention times [114,117,124] appears to influence nutritional status, oxidative stress and digestion processes in mussels. Alternatively, the massive presence of *I*. *galbana* within digestive cells may hamper the measurement of fluorescent-based cytochemical biomarkers and may bias biochemical biomarkers due to the high abundance of the algae in the mussel tissue. Interestingly, at low dietary cell concentrations of *I*. *galbana* (2×10^4^ cells/mL) the occurrence of microalgae within digestive cells is negligible [125]; however, rations over 2×10^4^ cells/mL are recommended and commonly used in physiological experiments [36, 126]. Further research is needed to optimize dietary food type, composition, regime and rations for toxicological experimentation. Meanwhile, it is important that research papers include a detailed description of the food type and feeding conditions to aid in comparison and interpretation of the biological responses elicited by pollutants in mussels.

## Supporting information

S1 Fig **Cytochrome-c-oxidase (COX) activity in gills (A) and protein carbonyl groups (CO) in digestive gland (B);** as recorded in mussels fed *ad libitum* for 1 week with 4 different diets (*I*. *galbana* (I); *T*. *chuii* (T); mixture of *I*. *galbana* and *T*. *chuii* (I+T); and commercial food (CF)).
(PDF)

S1 Table The effects of food type (d.f. = 3) on biomarkers in mussels fed *ad libitum* for 1 week with 4 different microalgae diets (*I. galbana; T.chuii; I. galbana* + *T. chuii* mixture; and commercial food). (PDF)
