# Supplementary material for: Food-type may jeopardize biomarker interpretation in mussels used in aquatic toxicological experimentation
Source: PLoS One. 2019 Aug 5;14(8):e0220661. doi: 10.1371/journal.pone.0220661 (PMC6681955; doi:10.1371/journal.pone.0220661)
Supplement: S1 Fig — Cytochrome-c-oxidase (COX) activity in gills (A) and protein carbonyl groups (CO) in digestive gland (B); as recorded in mussels fed ad libitum for 1 week with 4 different diets (I. galbana (I); T. chuii (T); mixture of I. galbana and T. chuii (I+T); and commercial food (CF)). (PDF) [file pone.0220661.s001.pdf]

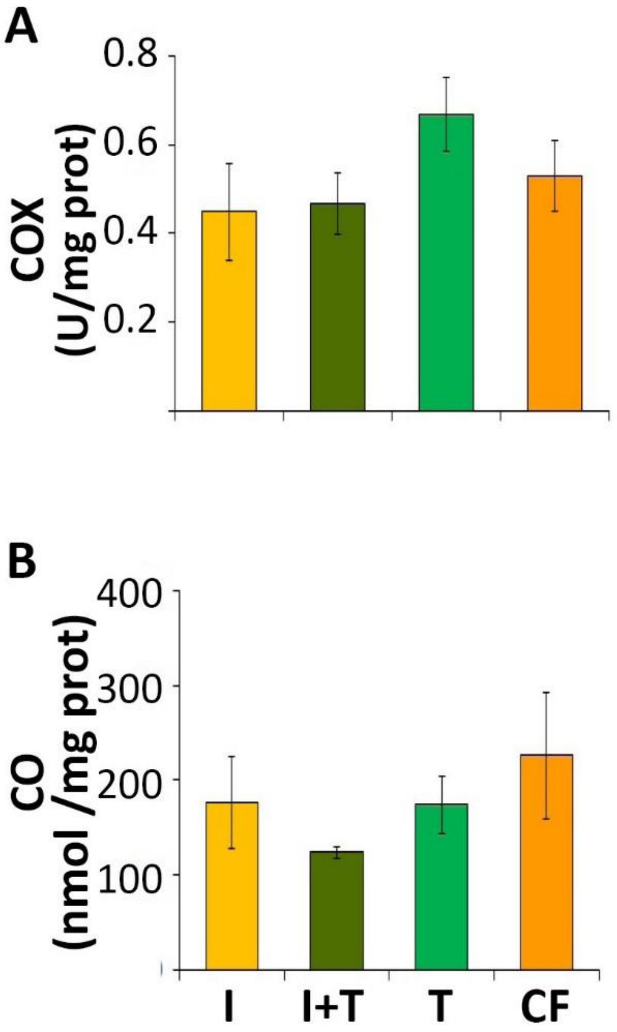

S1 Figure. Cytochrome-c-oxidase (COX) activity in gills (A) and protein carbonyl groups (CO) in digestive gland (B); as recorded in mussels fed *ad libitum* for 1 week with 4 different diets (*I. galbana* (I); *T. chuii* (T); mixture of *I. galbana* and *T. chuii* (I+T); and commercial food (CF)). Intervals indicate standard error.
