# Supplementary material for: Food-type may jeopardize biomarker interpretation in mussels used in aquatic toxicological experimentation
Source: PLoS One. 2019 Aug 5;14(8):e0220661. doi: 10.1371/journal.pone.0220661 (PMC6681955; doi:10.1371/journal.pone.0220661)
Supplement: S1 Table — (PDF) [file pone.0220661.s002.pdf]

S1 Table. The effects of food type (d.f. = 3) on biomarkers in mussels fed *ad libitum* for 1 week with 4 different microalgae diets (*I. galbana; T.chuii; I. galbana* + *T. chuii* mixture; and commercial food). d.f.: degrees of freedom; F: Fisher's F-ratio; X^2: chi-square; p: probability (significant: p<0.05). *Significant differences between diets according to the Z-score test (p<0.05; see Figs.).

	F	**d. f. (residual)**	**p**
**One-way ANOVA**			
**PK**	3.242	8	0.081
**PK/PEPCK**	2.267	8	0.158
**MDA**	2.093	6	0.203
**HNE**	4.021	12	0.034*
**$Vv_{LYS}^{\#}$**	3.857	9	0.050*
**$S/V_{LYS}^{\#}$**	1.647	9	0.247
**$Nv_{LYS}^{\#}$**	1.169	9	0.374
**Vv_{NL}**	6.696	11	0.008*
**Vv_{LPF}**	7.825	16	0.002*
**Vv_{BAS}**	5.256	16	0.041*
**MLR/MET**	3.429	16	0.043*
**Kruskal-Wallis**			
	**X^2**	**N**	**p**
**COX**	2.174	18	0.537
**PEPCK**	4.212	12	0.240
**CO**	1.471	16	0.689
**CTD ratio**	10.337	20	0.016*

PK: pyruvate kinase; PEPCK: phosphoenolpyruvate carboxykinase; MDA: malondialdehyde; HNE: 4-hydroxy-2-nonenal; Vv_{LYS}: lysosomal volume density; S/V_{LYS}: surface/volume of lysosomes; Nv_{LYS}: lysosomal numerical density; Vv_{NL}: volume density of neutral lipids; Vv_{LPF}: volume density of lipofuscins; Vv_{BAS}: volume density of basophilic cells; MLR/MET: mean luminal radius/mean epithelial thickness of the digestive alveoli; COX: cytochrome C oxidase; CO: protein carbonyl groups; CTD ratio: connective-to-diverticula ratio.
